# Under vaccination of children among Maasai nomadic pastoralists in Kenya: is the issue geographic mobility, social demographics or missed opportunities?

**DOI:** 10.1186/s12889-018-6309-5

**Published:** 2018-12-19

**Authors:** Anne M. Pertet, Dan Kaseje, Careena F. Otieno-Odawa, Lydia Kirika, Caleb Wanjala, Jackline Ochieng, Millicent Jaoko, Winnie Otieno, Dan Odindo

**Affiliations:** Tropical Institute of Community Health and Development, Great Lakes University of Kisumu, P.O Box 2224, 40100, Kisumu, Kisumu, Kenya

**Keywords:** Pastoral nomadism, Geographical mobility, Social demographics, Missed opportunities, Maasai, Under-vaccination, Severe under vaccination

## Abstract

**Background:**

Nomadic lifestyle has been shown to be a significant factor in low immunization coverage. However, other factors which might aggravate vaccination uptake in nomadic pastoralists are poorly understood. Our study aimed at establishing the relative influence of social demographics, missed opportunities, and geographical mobility on severe under vaccination in children aged less than two years living in a nomadic pastoralist community of Kenya.

**Methods:**

We used cross-sectional analytical study design. An interviewer-administered questionnaire was used to obtain quantitative data from 515 mothers with children aged less than two years. Under vaccination was the sum the total number of days a delayed vaccine was given after the recommended age range for each vaccine. Severe under vaccination was defined as those children who remained under-vaccinated for more than six months. Geographical mobility was assessed as household members who had gone to live or herd elsewhere in the previous 12 months*,* missed opportunity included questions on whether a child visiting a health facility had missed being vaccinated, while social demographic data included household size and mothers social demographics.

**Results:**

Three-quarters of the mothers had no formal education. One-third of the children had been taken to a health facility and missed being vaccinated. Forty percent of the households had moved in the previous 12 months. Prevalence of missed opportunity was 30.1%; 42.2% of children had not received any vaccines by their first birthday, and 24.1% of children were severely under vaccinated.

No significant association was found between social demographics and under-vaccination. Variables associated with under-vaccination were; movement of the whole family, (*p* = .015), missed opportunity, (*p* = <.001), lack of vaccines, (*p* = (.002), and location of health facility, (*p* = <.001). Movement of women and children made a significant contribution (*p* = 0.006) to severe under-vaccination. Children in households where women and children had moved were nine times more likely to be severely under-vaccinated than in those households where there was no movement.

**Conclusion:**

Geographic mobility of women and children was a key determinant of severe under vaccination among nomadic pastoralists in Kenya.

## Background

It is estimated that approximately 20 million pastoralists live in the Horn of Africa [1]. These pastoralists move seasonally in search of water, grazing lands, better livelihoods or simply to safer environments, [2]. This migration usually follows seasons and availability of resources such as water and pasture. Mobile pastoralists are virtually excluded from health services including immunization because the provision of health services including immunization services of children are not adapted to their way of life, [3].

Scientific studies have shown low vaccination coverage amongst the pastoralists. Oral poliovirus vaccine (OPV3) coverage of children aged less than 5 years in pastoralist communities was found to be much lower (28%) than children from settled communities in Kenya (85%), [4–6]. Nomadic lifestyle is also a significant factor influencing low immunization coverage, [5]. Another study in Kenya found that 60% of children from nomadic households had never been vaccinated compared to 7.2% of children from settled pastoralists. The mothers from nomadic pastoralists also had lesser knowledge of the importance of vaccination compared to mothers from settled households, [6]. This pattern of low coverage of vaccination in pastoralist communities when compared to settled communities was shown in other studies from Somalia, Mozambique, and Tanzania, [7–10]. Epidemiological studies suggest significant immunity gaps in the pastoralist population. All five wild poliovirus type one (WPV1) cases in Somalia occurred among nomadic-pastoralist families. Amongst these, four of the cases had not received any dose of polio vaccine, while one of them had only received one single dose, [11].

Social demographics such as maternal age, geographical location, education, religion, literacy, wealth index, marital status, occupation, knowledge of immunization, and distance to health facility have also been significantly associated with the use of immunization services and/or immunization coverage, [3, 10, 12–15]. Other studies have found socio-demographic variables such as sex of the child, maternal factors were not associated with defaulting [14, 16]. Older studies indicated cultural discrimination against female children, with boys having a greater chance to be vaccinated, [17].

Missed opportunities for vaccination is another factor associated with low immunization. Children who present for immunizations and receive immunizations on that day indicate good local immunization coverage among children accessing health facilities. Low missed opportunities for immunization at health facilities reflect good immunization coverage among children accessing health facilities. Variables contributing to a significant difference between children with and without missed opportunities for vaccination have shown that the quality of health services was an important cause of missed opportunities in a review of 79 missed opportunity studies, [18]. Descriptive analysis of private and public health facilities in Kenya, Malawi, Senegal and Tanzania revealed the following: few public or private providers assessed the child’s vaccination status during a sick child visit (range by country and facility type, 14–44%); very few providers discussed the importance of new-born vaccination, and few providers reported administering vaccinations or referred the child for vaccination during sick child visits. Providers indicated that: the child was not due for the vaccination, they had not checked the child’s vaccination status, or that the sick child visit occurred on a non-immunization day, [19]. Low missed opportunities for vaccination at health facilities of 4.6% were found in Cape Town, [20]. Results from Cape Town further indicated that having the Road-to-Health booklets (RTHBs) during consultation had the largest effect on immunization coverage. Another study in Uganda found higher (59.6%) missed opportunities for vaccination in children who sought treatment in a health facility, while 24.4% missed an opportunity during routine vaccination sessions. Children with missed opportunities for vaccination were also more likely to have an incomplete vaccination status than children without missed opportunities, [21].

Though empirical research shows that nomadic pastoralism has been associated with low vaccine uptake, we found no evidence of studies conducted on nomadic pastoralism which took into account missed opportunities for vaccination. Furthermore, vaccination studies on nomadic pastoralists have focused on coverage or completion, which present an accumulation of the required number of doses by infants irrespective of the timing of vaccine administration. We have found no studies in Africa based on under-vaccination. We, therefore, postulate that though nomadic pastoralism might lead to low vaccine uptake, missed opportunities and social demographics interact to increase under vaccination in children.

Our study aimed at establishing the relative influence of social demographics, missed opportunities, and geographical mobility on severe under vaccination in children aged less than two years living in a nomadic pastoralist community of Kenya.

## Methods

Data and methodology used in this paper were from a project titled ‘Community engagement through the Community Health strategy for the improvement of immunization coverage in Pastoral and nomadic communities in Kenya’ which was supported by International Initiative for Impact Evaluation (3ie) programme.

### Study site and population

This study was carried out in Magadi and Keekonyookie wards of Kajiado West constituency, Kajiado County, Kenya in February 2016. Kajiado West constituency has five administrative wards. Vaccination coverage of all vaccines except BCG fell below the recommended 90% in Kajiado County, [22]. There had been a severe drought in the County 12 months prior to our survey. Kajiado County, a nomadic pastoralism region is classified as high drought risk situation. The vegetation condition index (VCI) showed a VCI value close to 20 (< 20 is severe drought). Magadi ward had a VCI of 18.95 indicating severe drought situation, [23].

### Sampling

We used a cluster sampling technique. Magadi ward was purposively selected due to severe drought situation and cattle migration, while Keekonyookie which is neighbouring ward with less migration was also included in the study. Magadi has 12 sub-locations with a total population of 23,108, while Kekonyookie has a total population of 36,562 in nine sub-locations.

We began by listing all the villages in each of the sub-locations. Each village formed a primary sampling unit (PSU), while the households in the village were secondary sampling units (SSUs). The PSUs (villages) did not have the same population, therefore, we selected PSUs using Probability Proportional to Size sampling (PPS) which gave large PSUs a greater probability of occurring in the sample than small PSUs. All villages along with respective households and their populations were entered into the ENA software, [24]. The number of households to be studied in each village were selected accordingly. The first household in each village was selected by spinning a bottle to give the direction to follow. Households falling in this direction were visited until all mothers with eligible children were interviewed. Such children were recruited consecutively from the village until the sample size was realized. Within the selected households all mothers with children less than 24 months were interviewed. If there were two index children in the house, the youngest was selected.

### Sample size

Fisher’s formula, [25] *n* = z^2^pq/d^2^ was used to determine the sample size. n = desired sample size; Z = the abbisca of normal distribution (z = 1.96); p = the proportion of children who were fully immunized of 56.2% in the County, [22]; q = 1-P (proportion not fully immunized; d = maximum degree of error with a confidence interval of 95% = 0.05. This calculation gave a minimum sample size of 378 children. We adjusted this minimum sample size by multiplying it by 1.3 to take account of the cluster effect giving a sample size of 491. We also assumed an expected response of 75% (given this was a mobile community which gave a sample size of 504. The final sample size was 515.

### Study design and measurement of variables

We used cross-sectional analytical study design to explore the association between the outcome, (under- vaccination) and explanatory variables with adjustment for potential confounding variables. Age the child was vaccinated was calculated from the interval between the birth date and the date on the vaccination card. Most vaccinations were dated in the health cards. Where vaccinations were registered without a date, we estimated the age of the child from the mother. Recommended vaccines to be given at specific ages were based on the Kenya Expanded Program on Immunization, [26]. Vaccine *completion* was defined as the accumulation of the required number of doses by infants by their first birthday irrespective of the timing of vaccine administration. Under vaccination was assessed by *summing the total number of days a delayed vaccine was given after the recommended age range* for each vaccine per child. If a dose was never received by 12 months, the child received the maximum number of days under-vaccinated *based on the total number of days between the first day of under-vaccination and age 12 months*. Severe under vaccination was defined as those *children who remained under vaccinated for more than six months*. This approach has been used in other studies, [26–28].

Geographical mobility was assessed by questioning the respondent on whether any member of the *household had gone to live or herd somewhere in the last 12 months, and which members of the family members went.*

A missed opportunity is an occasion when a person eligible for immunization and with no valid contraindication visits a health service facility and does not receive the vaccine, [18]. Missed opportunity included questions on whether the child was taken to a health facility in the previous 1 month; the reason for taking the child to the health facility; whether the mother was asked if the child had been vaccinated; whether she had taken the child to the clinic and the child was not vaccinated, and the reasons given for not vaccinating the child by the health staff. The mother was also asked whether she knew why the child needed to be vaccinated.

Data were also collected on social demographics such as household size (number), mother’s education, occupation, age, marital status, and occupation.

### Survey instrument

An interviewer-administered questionnaire was used to obtain the data. The instrument included variables on socio-demographics, missed opportunities, geographical mobility, and mother’s knowledge of why children are vaccinated. All questions in the survey instrument were close-ended. In addition, information about the child’s vaccination was obtained from the vaccination card.

Data collection and management: Data quality was ensured through onsite supervision and review of completed forms. Each completed survey form was reviewed for completeness prior to analysis by the supervisors. Data were entered using Statistical Package for the Social Sciences (SPSS), version 20 for Windows data file. Double data entry was performed. Data were cleaned and several consistency checks carried out to ensure accuracy and completeness. Data checking and cleaning methods included examining ranges of responses for each variable through frequency distributions for possible oversight upon entry, normality, and outliers. Missing data were addressed through System-missing for blank cells using − 1 value for missing blank values. The advantage of this approach is that the process produced correct relationship matrices. Fisher test of skewness was used to assess whether or not the continuous data were normally distributed.

### Data analysis

This analysis was based on children who were less than 24 months and who had a vaccination card at the time of the interview. Subjects whose ages were outside the eligible age group were excluded from data analysis. ‘Though cluster sampling effect was taken into account during the sample size calculation, weighting factors from the sample were not used to analyse the data. Both descriptive and inferential statistics were computed. Frequency distributions and percentages were used to summarize key study variables, and simple frequency tables of all the variables were made. Total scores were created for overall under vaccination before statistical inferences were made. Under vaccination was then categorized as under-vaccinated (i.e under-vaccinated for less than including 6 months), and severely under-vaccinated (i.e under-vaccinated for more than 6 months). Counts and proportions were used to summarize categorical variables. Chi-square statistic was used to test whether there were significant differences in the proportion of children who were under-vaccinated and those who were severely under-vaccinated by geographical mobility, missed opportunities and social demographics. Prevalence Odds Ratio (OR) and corresponding 95% Confidence Interval (CI) were used. Variables that were found to be significant at *p* < 0.05 from the bivariate analysis were entered into a binary logistic regression model in order to investigate relative effect of the explanatory variables in relation to the dependent variable and any confounding between them. The explanatory variables were geographical mobility, missed opportunities and social demographics, while the response variable was under-vaccination. Decisions for the statistical significance of the findings were made using an alpha level of < 0.05.

SPSS version 20 logistic regression tool was used to compute a binary logistic regression which showed the odds probability of belonging to either group (the severely under-vaccinated or under-vaccinated). A model (i.e. an equation) was created that included variables that were useful in explaining under-vaccination. These variables included: movement of the whole family, a child taken to the hospital and not vaccinated, no vaccines, and the location of the health facility. All these variables were entered at the same time providing only one model. The model was applied to data where the dependent variable (under vaccination) was categorical. The composite variable predicted the probability of a case being in the severely under-vaccinated group.

### Ethical considerations

Approval for the study was granted by the Great Lake’s University of Kisumu. Caregivers provided verbal consent for participation after the research assistants explained to the participants that their involvement was voluntary and that they were free to withdraw from the study at any time, *without giving a reason*. The respondents could also refuse to answer questions they were uncomfortable with, and their data could be removed from the study if they so wished. The study participants were assured of anonymity and confidentiality of the information they provided and were also assured that they would not be identified by name but by an identification number. The consent was mostly oral or thumb printed. No incentives were provided to take part in the study.

## Results

Descriptive statistics: Frequency distribution of social demographics are summarized in Table 1. Data was collected from 515 children who had vaccination cards. The sample had slightly more males (52.8%) than females (47.2%). Majority of the mothers were aged below 25 years (58.1%) and most had no formal education (74.5%). Regarding literacy level, only 31.8% of the mothers could read; 29.0% could write, while 28.8% had the ability to read and write. Caretakers were mainly housewives, while household heads were herders, (77.2%). The number of household members averaged 6 and ranged from 1 to 12.

**Table 1:** Socio-Demographics Characteristics of the Mothers and children (*n = 515)*

Variable	Percent
Caretaker’s Age in years Mean age	25.38 sd ±6.90
Less than 20 years	26.3
21–25 years	31.8
26–29 years	19.0
Over 29 years	22.9
Marital status	
Single	5.9
Married	94.1
The education level of household head	
No Formal Education	74.5
Primary education	18.0
High school	7.4
The education level of the mother/caretaker	
No Formal Education	74.1
Primary education	21.4
High school	4.4
Literacy level	
Ability to read	31.8
Ability to write	29.0
Ability to read and write	28.8
Age of the child in months	
Less than one year	42.8
12–23 months	48.9
Over 23 months	8.3
Mean age of children	51.3 ± 28.9
Occupation of the caregiver	
Employed	1.2
Self-Employed	7.8
Housewife	69.9
Casual Labourer	3.2
Livestock Farmer	17.8
The main source of income	
Skilled Labour	12.8
Herder	77.2
Farmer	10.0

### Health-related background factors

Table 2 describes health-related factors. Knowledge of why children are vaccinated, visit by community health workers and sources of information had multiple responses, and thus do not add to 100%. While 89.9% of the mothers had attended antenatal care (ANC), only 36.1% had attended ANC for the recommended four times. Few children were delivered by a health practitioner (20.6%). Most of the babies were delivered at home (82.2%), with 50.9% being conducted by a Traditional Midwife.

**Table 2:** Health-related factors *(n = 515)*

Variable	Percent
The person who makes decisions on child vaccination	
Father	43.2
Mother	35.5
A consensus of both parents	18.7
Other relatives	2.6
Means of transport to the health facility	
Walk	66.7
Bicycle/Motorcycle	21.2
Motor vehicle	12.0
Attendance of antenatal care	
None	10.1
Once	10.4
Twice	12.5
Thrice	21.9
Four times	36.1
More than four times	9
The person assisting in the delivery of child	
Health staff	20.6
Relative friend	27.3
Traditional midwife	50.7
Self-delivered	1.0
Evidence of BCG scar	87.1
Knowledge of why children are vaccinated	
To prevent diseases	82.0
Health growth of children	18.0
Cure diseases	8.9
Not sure	8.2
Visit by community health worker	74.1
To give health education	39.6
Talk on immunization	50.6
Collect data	7.1
Heard messages on vaccination	86.1
Sources of information	
Radio	32.0
TV	3.5
Newspaper	3.6
Health facility	55.6
Telephone message	3.4
School	5.7
Church	8.6
Community health worker	54.0

The average time taken to walk to the health facility was about 2 hours, (mean 108 ± 78 min, median 90 min, and mode 120 min). Most caregivers (66.7%) walked to the health facility.

Three quarters (74.1%) of the households had been visited by a community health worker in the previous 12 months. The main purpose of the visit (50.6%) was provision of information on immunization to mothers. Other sources of information on immunization were health facilities and radio. Many mothers 82.0% knew that immunizations prevent diseases and children can get diseases if not vaccinated.

About 30.1% of the mothers reported that they had taken their child to the health facility and the child was not vaccinated (missed opportunity). The main reason given for missed opportunity by the health facility staff were lack of vaccines (24.3%).

### Geographical mobility and missed opportunities

Table 3 gives descriptive results of the geographical mobility and missed opportunity. The results show that though 57.8% children had completed vaccination by the age of two years, only 2% of children had received all vaccinations at the recommended times. Forty-two percent (42.2%) of children had not received any vaccines by their first birthday. Approximately 24.1% % of children remained under-vaccinated for more than six months (severe under vaccination).

**Table 3:** Geographical mobility and missed opportunity *(n = 515)*

Variable	Percent
Number of vaccines completed by first birthday	
None	42.2
One	15.7
Two	9.0
Three	8.4
Four	12.5
Five	10.6
Six	1.4
Seven	0.2
Severely under vaccinated (more than six months)	24.1
Geographical mobility	
Yes**/**Household moved	40.2
Number of times family moved	
Once	15.5
Twice	16.1
More than twice	12.0
No response	6.6
Person moving	
The whole family	54.6
Adult males	33.0
Women and children	12.3
Missed opportunities	
The child was taken to a health facility in the last one month	63.6
Reason for taking the child to a health facility	
The child was sick	51.2
Clinic day	31.0
No response	17.8
Asked whether the child had been vaccinated	55.1
Taken the child to a health facility and not vaccinated	30.1
Reasons given for not vaccinating the child	
Health staff said the child was not due for vaccination	9.4
There were no vaccines	25.5
Not a vaccination day	13.4
No reason was given	7.5

Forty percent (40%) of the households had moved in the previous 12 months. In most of the households (54.6%) the whole household moved, only 12.3% of women and children moved.

About 2/3rd of the mothers had taken their children to a health facility in the previous one month. Half (51.2%) of the children were taken to the facility due to sickness, while 31% were taken to the child health clinic. Only 55% of the mothers who visited the health facility were asked whether their child had been vaccinated. Missed opportunities for vaccination were found 30.1%. Lack of vaccines in the health facility was reported by 25.5% of the mothers.

### Factors associated with under vaccination

Variables contributing to a significant difference between children who were under-vaccinated and those who were severely under-vaccinated are examined in this section. Social demographics such as mothers’ age, marital status, schooling level, sex of the child, and understanding of the importance of vaccination showed no significant differences in respect to under-vaccination. Variables which were significantly associated with under vaccination using chi-square test were: movement of the whole family (*p* = .015); child taken to the health facility and not vaccinated (missed opportunity) (*p* = <.001); no vaccines, (*p* = .002), the location of the health facility (*p* = <.001).

SPSS version 20 logistic regression tool was used to compute a binary logistic regression which showed the odds probability of belonging to either group (severely under-vaccinated or under-vaccinated). A model (i.e. an equation) was created that included variables that were useful in explaining under-vaccination. Only variables which were significant from bivariate analysis were entered into the logistic regression. These variables included: movement of the whole family, a child taken to the hospital and not vaccinated, no vaccines, and the location of the health facility. All these variables were entered at the same time providing only one model. The model was applied to data where the dependent variable (under vaccination) was categorical. The composite variable predicted the probability of a case being in the severely under-vaccinated group.

Table 4 gives the results of binary logistic regression analysis. With exception of the movement of the household all variables found significant in the bivariate analysis lost their statistical significance when included in the binary regression logistic model and were thus considered confounders. The Wald criterion demonstrated that household movement (*p* = 0.003) made a significant contribution to severe under-vaccination. The movement of whole families or adult males did not make a significant contribution to severe under-vaccination. On the other hand, the ‘odds ratio’ where women and children moved had a coefficient of 9.0 and a 95% confidence interval of [1.11, 72.66]. This suggests that households where mothers and children moved, were 9 times more likely to be severely under-vaccinated than those in the households who did not move.

**Table 4:** Bivariate logistic regression analysis: Odds Ratios (OR), confidence intervals (95% CI) and significance

Explanatory variables	Significance	(OR)	95% CI for OR	
			Lower	upper
Family moved	0.003			
Whole family moved	0.295	2.75	.415	18.21
Only adults moved	.482	1.935	.307	12.214
Women and children moved	.039	8.99	1.11	72.66
No vaccine	.038	.643	.423	.977

Variable(s) entered on step 1: Family moved, no vaccine

Measures of log likelihood which are tentative indicators of the range of which the actual influence of the explanatory variables on the dependent variable lay, showed that the explanatory variables explained only 5 to 7% of the variation in the dependent variable using Cox & Snell R^2^ and Nagelkerke R^2^ respectively.

## Discussion

Interpretation of results: Our study shows that nomadic pastoralist who were mainly of Maasai origin, with low education and literacy levels maintained reasonable contact with modern health facilities. This is shown by their frequent visits to a health facility for antenatal care, sickness, and child health clinics. They walked long distances to benefit from these health services. We observed that mothers tended to move in groups to health facilities during child clinic days. Accessibility in terms of time taken to a health facility was not associated with under-vaccination. However, despite the long walks, our study reveals a high prevalence of missed opportunities for vaccination at the selected health facilities. Majority of children who presented specifically for immunizations did not receive immunizations, suggesting poor immunization coverage. The fact that health workers did not inquire whether the child had been vaccinated indicates that vulnerable children could have been missed. Vaccine stock-outs occurrence also worsened the situation. Differences observed on under vaccination across health facilities could have been due to their geographical location. Those reporting severe under vaccination were located in Magadi where the community was more mobile.

There was adequate information on immunization in this community. Community Health Workers (CHWs) were a potential source for disseminating immunization information relating to the immunization. Traditional birth attendants played a major role in delivering of infants.

Context: Contributory factors such as socioeconomic status and level of education did not play a significant role in under-vaccination. Though in the Maasai culture females are discriminated against socially, we found no differences in under vaccination by sex of the child. Our results conflicted with findings from other studies where boys had a greater chance of being vaccinated, [17]. Marital status and age of the mothers were also not associated with under vaccination in our study. These findings were consistent with those from other studies where the use of immunization services was not associated with marital status or age of the mother, [25]. In our study, the reason why no associations were found might have been that virtually all the mothers were married. In other settings, the age of the mother was associated with incomplete vaccination, [12, 14]. Low educational level of mothers has been associated with low vaccine uptake, [14, 15, 25], however, we found no association between education and under-vaccination. The explanation of why mothers’ educational levels had no influence on the child’s vaccination status, was probably because a majority of the mothers had limited education.

Previous studies in Mozambique have identified missed opportunities for vaccination as important factors inhibiting better EPI coverage, [8]. It has been reported that the quality of health services was an important cause of missed opportunities for vaccination, [18]. A large number of missed opportunities for vaccination in our study proved that the use of health services was high, probably reflecting poor immunization coverage among children accessing health facilities. This is contrary to studies from South Africa where missed opportunities for immunization at health facilities in Cape Town were low, probably reflecting good immunization coverage among children accessing health facilities, [20]. The differences in these results could be due to better health services in South Africa.

### Strength and limitations

One of the greatest strengths of our study was that it was household-based, thus, identifying missed opportunities at the household level which is more representative than studies conducted in health facilities. Furthermore, most literature on immunization focuses on coverage or completion while we focused on under vaccination which gives a total sum of days a child remained under-vaccinated. We have also come up with empirical results showing that the movement of women and children influences under vaccination more than the movement of whole families or adult males among nomadic pastoralists. However, the study has some weaknesses. Some essential data explaining missed opportunities for vaccination were missed. Since our study was cross-sectional we could not assess the number of missed opportunities for vaccination per child. We missed data on whether the health worker requested to check other information and whether mothers were aware of the need to bring the card at every visit. We also did not study the health facility factors such as staff attitudes towards immunization, heavy workload, pharmacy stock practice, lack of training and adherence to immunization guidelines. Weighting factors from the sample were not used to analyse the data, though they have important implications in resolving unbiased estimates.

## Conclusions

Geographic mobility of women and children were key determinants of severe under vaccination among nomadic pastoralists in Kenya. However, these variables accounted for only a small variation in severe under-vaccination, thus there is need for further studies with larger samples using mixed methodology (qualitative and quantitative) to find more answers to this phenomenon.

## Abbreviations

3ie: International Initiative for Impact Evaluation
BCG: Bacillus Calmette-Guérin
CHWs: Community Health Workers
CI: Confidence Interval
OPV: Oral poliovirus vaccine
PPS: Population proportional to size
PSU: Primary sampling unit
RTHBs: Road-to- health booklets
SPSS: Statistical Package for the Social Sciences
SSUs: Secondary sampling units
VCI: Vegetation condition index
WPV: Wild poliovirus type
